# Detection and characterization of eravacycline heteroresistance in clinical bacterial isolates

**DOI:** 10.3389/fmicb.2024.1332458

**Published:** 2024-03-27

**Authors:** Yingfeng Zhang, Dongdong Liu, Yongzhu Liu, Qiwei Li, Hongwei Liu, Peng Zhou, Yaqin Liu, Lili Chen, Weiguo Yin, Yang Lu

**Affiliations:** ^1^Department of Public Health Laboratory Sciences, School of Public Health, Hengyang Medical School, University of South China, Hengyang, Hunan, China; ^2^Department of Laboratory Medicine, Affiliated Qingyuan Hospital, Guangzhou Medical University, Qingyuan People’s Hospital, Qingyuan, China; ^3^Department of Laboratory Medicine, The Second Affiliated Hospital of Guangzhou University of Chinese Medicine, Guangzhou, China; ^4^Department of Gynecology, Affiliated Qingyuan Hospital, Guangzhou Medical University, Qingyuan People’s Hospital, Qingyuan, China

**Keywords:** eravacycline, heteroresistance, carbapenem-resistant *Klebsiella pneumoniae*, whole-genome sequencing, time-killing assay, fitness cost

## Abstract

Eravacycline (ERV) has emerged as a therapeutic option for the treatment of carbapenem-resistant pathogens. However, the advent of heteroresistance (HR) to ERV poses a challenge to these therapeutic strategies. This study aimed to investigate ERV HR prevalence among common clinical isolates and further characterize ERV HR in carbapenem-resistant *Klebsiella pneumoniae* (CRKP). A total of 280 clinical pathogens from two centers were selected for HR and analyzed using population analysis profiling (PAP) and modified *E*-tests. The PAP assay revealed an overall ERV HR prevalence of 0.7% (2/280), with intermediate heterogeneity observed in 24.3% (68/280) of strains. The proportion of heteroresistant strains was 18.3% according to modified *E*-test results. A time-killing assay demonstrated that CRKP CFU increased significantly after 10 h of ERV treatment, contributing to the reduced bactericidal effect of ERV *in vitro*. Interestingly, dual treatment with ERV and polymyxin B effectively inhibited the total CFU, simultaneously reducing the required polymyxin B concentration. Furthermore, fitness cost measurements revealed a growth trade-off in CRKP upon acquiring drug resistance, highlighting fitness costs as crucial factors in the emergence of ERV HR in CRKP. Overall, the findings of the current study suggest that ERV HR in clinical strains presents a potential obstacle in its clinical application.

## Introduction

*Klebsiella pneumoniae* (*K. pneumoniae*) has been acknowledged as an important opportunistic pathogen responsible for a multitude of hospital-acquired infections (Wyres et al., 2020; Wang et al., 2022). The rising incidence of carbapenem-resistant *K. pneumoniae* (CRKP) infections has garnered global attention, underscoring the pressing need for more effective antibiotics. Colistin, considered the last line of defense against bacterial infections, has demonstrated satisfactory activity against CRKP *in vitro* (Bialvaei and Samadi Kafil, 2015). Nevertheless, with rising colistin use, colistin resistance has become a major concern (Carfrae et al., 2023). With evidence indicating that CRKP could render colistin treatments ineffective, eravacycline (ERV) could be considered a last-resort drug for the treatment of CRKP infections, especially in countries where colistin is not commercially available.

ERV, a novel fluorocycline antibiotic, is a fourth-generation tetracycline introduced after tigecycline (Blanchard et al., 2023). It acts as an antimicrobial agent by reversibly binding to the bacterial ribosomal 30S subunit, thereby preventing amino acid residues from being incorporated into elongating peptide chains, which leads to the disruption of bacterial protein synthesis (Zhanel et al., 2016). *In vitro* studies have validated its broad-spectrum activity against aerobic and anaerobic gram-positive and gram-negative pathogens, including methicillin-resistant *Staphylococcus aureus* (MRSA), extended-spectrum beta-lactamase (ESBL)-producing *Enterobacteriaceae*, carbapenem-resistant *Enterobacteriaceae*, and multidrug-resistant *Acinetobacter baumannii* (*A. baumannii*) (Scott, 2019). In addition, studies reported that ERV is 2–4 times more effective than tigecycline against clinically prevalent aerobic bacteria (Scott, 2019). Despite the promising potential of ERV, the challenge of ERV heteroresistance (HR) cannot be disregarded.

HR denotes the phenomenon where different subpopulations of bacteria with identical genetic backgrounds exhibit a range of antibiotic sensitivities (El-Halfawy and Valvano, 2015; Abbott et al., 2023; Doijad et al., 2023). Even though this phenomenon has been observed for decades, it has yet to receive sufficient attention in clinical research. Alarmingly, clinically common pathogens such as *Escherichia coli* (*E. coli*) (Nicoloff et al., 2019; Diaz-Diaz et al., 2023), *K. pneumoniae* (Zheng et al., 2018; Sánchez-León et al., 2023), *Staphylococcus aureus* (*S. aureus*) (Bai et al., 2019), *A. baumannii* (Jo and Ko, 2021; Jo et al., 2023), and *Pseudomonas aeruginosa* (*P. aeruginosa*) (Choby et al., 2021) exhibit high rates of antibiotic HR, which could potentially increase the incidence of infections and mortality rates. The mechanism of HR is complex, with literature suggesting that the high prevalence of antibiotic HR is predominantly attributed to spontaneous amplification of resistance genes (Nicoloff et al., 2019). Additional contributing factors include point mutations, insertion sequences (IS), insertions or small deletions, overexpression of antibiotic resistance genes, reduced expression of porin protein-encoding genes, and biofilm formation (Adams-Sapper et al., 2015; Nicoloff et al., 2019; Chen et al., 2020).

While ERV HR has been detected in *S. aureus* (Zhang et al., 2018) and *K. pneumoniae* (Zheng et al., 2018), the specific mechanisms driving this resistance remain elusive. Additionally, ERV HR in other common clinical pathogens has yet to be documented. Considering the limited clinical application of ERV in China, investigating ERV HR prevalence is imperative. Hence, this study aimed to evaluate the prevalence of ERV HR in common clinical pathogens across two centers and investigate the related HR mechanisms in CRKP. The insight gained is anticipated to facilitate a deeper comprehension of the mechanisms underlying ERV HR and amplify vigilance toward CRKP treatment with ERV, thus supporting the judicious use of antibiotics in clinical practice.

## Materials and methods

### Bacterial strains and antibiotic susceptibility testing

In the present study, a total of 280 clinical isolates collected from two regions between 2018 and 2022 were examined. Of these, 183 strains originated from the Sixth Affiliated Hospital of Guangzhou Medical University, comprising 15 (8.2%) strains of carbapenem-resistant *E. coli* (CREC), 34 (18.6%) strains of MRSA, 37 (20.2%) strains of *Enterococcus faecalis* (*E. faecalis*), 44 (24.0%) strains of ESBL-producing *E. coli* (eco-ESBL), 16 (8.7%) strains of *Enterobacter cloacae* (*E. cloacae*), 23 (12.6%) strains of *K. pneumoniae*, and 14 (7.7%) strains of *P. aeruginosa*. The remaining 97 strains were sourced from the Second Affiliated Hospital of Guangzhou University of Chinese Medicine, including 16 (16.5%) strains of CREC, 30 (30.9%) strains of MRSA, 29 (29.9%) strains of carbapenem-resistant *A. baumannii* (CRAB), and 22 (22.7%) strains of CRKP. Each strain was identified using matrix-assisted laser desorption/ionization time-of-flight mass spectrometry (MALDI-TOF MS). CR, ESBL, or methicillin-resistant (MR) strains were identified using the BD Phoenix™ M50 instrument. The clinical isolates were preserved at −80°C, and prior to each experiment, they were revived and cultured on fresh blood agar plates. The minimum inhibitory concentration (MIC) of antibiotics was ascertained using the standard broth microdilution method. Susceptibility data for ERV and polymyxin B (PMB) were interpreted according to the European Committee on Antimicrobial Susceptibility Testing (EUCAST) guidelines. However, since the EUCAST *A. baumannii* MIC breakpoint for ERV remains undefined, the thresholds from a reference study (Zhanel et al., 2018) were adopted: ERV susceptible at ≤0.5 mg/L and ERV resistant at >0.5 mg/L.

### Population analysis profiling (PAP)

A modified PAP method was employed to detect ERV HR (Nicoloff et al., 2019). Briefly, bacterial strains were inoculated in LB broth and incubated overnight at 37°C. Then, 10 μL of the bacterial suspension was transferred into 2 mL of LB broth and incubated until the logarithmic growth phase (6 h) was reached. The concentration of the bacterial solution was adjusted to 10^7^ CFU/mL using LB broth. Subsequently, 20 μL of the bacterial solution was evenly spread on PAP gradient antibiotic plates, which were then incubated at 37°C for 48 h. After incubation, the number of colonies on each plate was recorded and plotted against the concentration of ERV using GraphPad Prism 8 software. The frequency of the heteroresistant subpopulation was calculated by dividing the number of colonies on plates with the highest antibiotic concentration by the number of colonies on antibiotic-free plates (Zhang et al., 2021). If the ratio of the MIC to the highest non-inhibitory concentration (HNIC) exceeded 8, the strain was defined as ERV HR-positive. Intermediate heterogeneity was designated when the MIC/HNIC ratio was 8 (El-Halfawy and Valvano, 2015).

### Modified *E*-tests

The modified *E*-tests employed cell densities higher than the standard to enhance the presence of resistant bacteria from the subpopulation (Wootton et al., 2001). Adhering to the methodology established by Nicoloff et al. (2019), bacterial colonies cultured overnight in Mueller-Hinton broth (MHB) were adjusted to 2 MacFarland units. The adjusted sample was uniformly spread across an M-H agar plate using sterile cotton swabs, followed by the placement of *E*-test strips. After a 48-h incubation at 37°C, the outcomes were observed. The generally accepted notion is that heteroresistant strains can proliferate within the inhibitory zone, whereas non-heteroresistant strains cannot.

### Time-killing assay

Selected clinical isolates were subjected to time-killing assays. A single colony was inoculated into LB broth and incubated overnight at 37°C to facilitate bacterial proliferation. Subsequently, 10 μL of the bacterial suspension was transferred into 2 mL of MHB. After reaching the logarithmic growth phase (6 h), the bacterial concentration was adjusted to 10^6^ CFU/mL using MHB. The MHB was then supplemented with 1x MIC ERV concentration (or 16 mg/L PMB or 1x MIC ERV with 4, 8, 16 mg/L PMB) and incubated in a shaking incubator set at 37°C and 210 rpm. Culture samples (10 μL each) were periodically extracted at 0, 4, 6, 8, 10, 12, and 24 h, serially diluted, spread on antibiotic-free nutrient agar plates, and incubated at 37°C for 48 h for subsequent colony counting. Statistical analysis and plotting were performed using GraphPad Prism software 8.0.

### Antibiotic resistance stability and fitness cost measurements

The stability of antibiotic resistance in CRKP3 was tested using the PAP method. CRKP3 subpopulations were isolated on PAP plates with a 0.5x MIC concentration. Recovery bacteria were obtained as follows: single cell clones were isolated on PAP plates at a 0.5x MIC concentration (CRKP3 subpopulations), and a single colony was inoculated into 3 mL of LB broth with ERV (antibiotic concentration at 0.5x MIC), then incubated overnight at 37°C and 210 rpm. Thereafter, 3 μL of the bacterial suspension was transferred into 3 mL of fresh antibiotic-free LB broth and incubated under the same conditions for a 24-h period. This antibiotic-free culture process was repeated for 10 days to obtain recovery bacteria. The heteroresistant subpopulations and recovery bacteria of CRKP3 were analyzed using the PAP method, and the findings were compared to those of the parental CRKP3 strain.

For fitness cost assessments, the CRKP3 parental strain, subpopulations, and recovery bacteria were revived on nutrient agar plates. A single colony was inoculated into 3 mL of LB broth. The parental strain and recovery bacteria were cultured in the absence of antibiotics, while the LB broth for the subpopulations contained ERV (antibiotic concentration at 0.5x MIC). The cultures were incubated overnight at 37°C and 210 rpm. Thereafter, 3 μL of bacterial suspension was transferred to 3 mL of LB broth and incubated under the same conditions for 8 h. The bacterial suspension was then diluted 1:100 with LB broth and incubated at 37°C and 210 rpm for 24 h. Absorbance at 600 nm for three replicates of the bacterial medium was measured every 2 h. Bacterial growth curves were plotted using GraphPad Prism software 8.0.

### Whole-genome sequencing (WGS) analysis

Genomic DNA was extracted using a DNA extraction kit (TIANGEN, Beijing, Co Ltd.). The quality of the DNA was assessed via agarose gel electrophoresis, while quantification was achieved using the Qubit® 2.0 Fluorometer. The whole genomes of the CRKP parental strain, subpopulation, and recovery bacteria were sequenced using Illumina NovaSeq PE150 platforms (Novogene Bioinformatics Technology Co., Ltd. Beijing, China). Raw sequence data were filtered to obtain high-quality data. These data were compared with the *K. pneumoniae subsp. pneumoniae* HS11286 reference sequence (NCBI accession number: NC_016845) using BWA software (v0.7.17). Variants were functionally annotated using ANNOVAR (Chen et al., 2009).

### Quantitative reverse transcription PCR analysis

Expression levels of efflux components, namely OqxB, MacA, AcrAB-TolC, along with the genes of the PmrAB and PhoPQ systems (*phoP*, *phoQ*, *pmrA*, *pmrB*) in the CRKP3 parental strains and subpopulations, were evaluated via quantitative real-time PCR (qRT-PCR). Details of the primer used for the qRT-PCR analysis are provided in Supplementary Table S1. Total bacterial RNA was extracted by the Trizol method, following the manufacturer’s instructions. cDNA was synthesized using the *Evo M-MLV* RT Premix (ACCURATE BIOTECHNOLOGY, Hunan, China), and gene expression was quantified using the QuantStudio 5 real-time PCR system (Applied Biosystems, Foster City, CA, United States). The housekeeping gene *rpsL* served as the internal reference. Relative quantification of target genes was performed with the 2^−ΔΔCt^ methodology.

### Statistical analysis

Statistical evaluations were conducted with GraphPad Prism software version 8.0. Measurement data were compared using the Student’s *t*-test and are presented as x ± s. Count data were analyzed using the chi-square test and are presented as n (%). *P* < 0.05 was deemed statistically significant.

## Results

### Antibiotic susceptibility results

The susceptibility of the 280 clinical isolates to ERV is detailed in Table 1. Among these isolates, 181 (64.6%) were susceptible to ERV, while 99 (35.4%) exhibited resistance. Briefly, 9 (29%) strains of CREC, 14 (21.9%) strains of MRSA, 7 (24.1%) strains of CRAB, and all 22 (100%) strains of CRKP were resistant to ERV. Intriguingly, all 44 (100%) strains of eco-ESBL were susceptible to ERV. Noteworthily, 19 (82.6%) strains of carbapenem-sensitive *K. pneumoniae*, 7 (18.9%) strains of *E. faecalis*, and 7 (43.8%) strains of *E. cloacae* were resistant to ERV. In addition, all 14 (100%) strains of *P. aeruginosa* exhibited resistance to ERV, aligning with previous findings indicating that *P. aeruginosa* isolates are not susceptible to ERV (Scott, 2019).

**Table 1:** Eravacycline susceptibility characteristics among 280 clinical isolates.

Bacteria	Strains	Eravacycline MIC (mg/L)	
		S^a^	R^b^
CREC	31 (100)	22 (71)	9 (29)
MRSA	64 (100)	50 (78.1)	14 (21.9)
CRAB	29 (100)	22 (75.9)	7 (24.1)
CRKP	22 (100)	0 (0.0)	22 (100)
eco-ESBL	44 (100)	44 (100)	0 (0.0)
*K. pneumoniae* ^c^	23 (100)	4 (17.4)	19 (82.6)
*E. faecalis*	37 (100)	30 (81.1)	7 (18.9)
*E. cloacae*	16 (100)	9 (56.3)	7 (43.8)
*P. aeruginosa*	14 (100)	0 (0.0)	14 (100)
Total	280 (100)	181 (64.6)	99 (35.4)

Data shown as n (%).

a S: Clinical strains were susceptible to eravacycline.

b R: Clinical strains were resistant to eravacycline.

c *K. pneumoniae*: carbapenem-sensitive *K. pneumoniae*.

### Prevalence of ERV HR in clinical isolates

**Figure 1:**
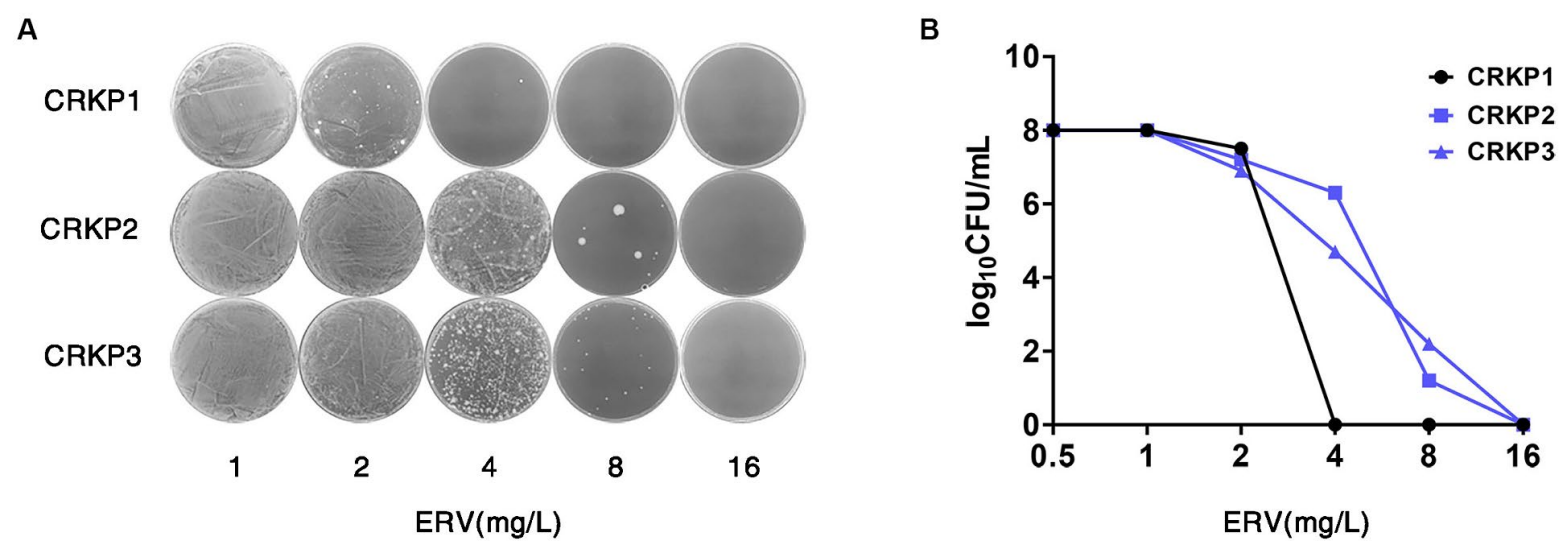
Screening and confirmation of ERV HR in CRKP strains. **(A,B)** PAP of the ERV-heteroresistant CRKP isolates. CRKP1 belongs to the NHR strain, and CRKP2 and CRKP3 belong to the heteroresistant strains. HR, heteroresistance; NHR, non-heteroresistant.

PAP was employed to assess 280 bacterial strains from two clinical centers in Southern China. The findings are delineated in Table 2. Among the evaluated isolates, 8 (25.8%) strains of CREC, 4 (6.3%) strains of MRSA, 9 (31.0%) strains of CRAB, 15 (68.2%) strains of CRKP, 17 (38.6%) strains of eco-ESBL, 7 (30.4%) strains of *K. pneumoniae*, 5 (13.5%) strains of *E. faecalis,* and 3 (18.8%) strains of *E. cloacae* exhibited intermediate heterogeneity to ERV. Furthermore, 2 (0.7%) strains of CRKP were identified as heteroresistant to ERV (Figures 1A,B).

**Table 2:** The distribution of heteroresistant bacteria.

Bacteria	Strains	I-HR strains	I-HR rates	HR strains	HR rates
CREC	31	8	25.8%	0	0.0%
MRSA	64	4	6.3%	0	0.0%
CRAB	29	9	31.0%	0	0.0%
CRKP	22	15	68.2%	2	9.1%
eco-ESBL	44	17	38.6%	0	0.0%
*K. pneumoniae* ^a^	23	7	30.4%	0	0.0%
*E. faecalis*	37	5	13.5%	0	0.0%
*E. cloacae*	16	3	18.8%	0	0.0%
*P. aeruginosa*	14	0	0.0%	0	0.0%
Total	280	68	24.3%	2	0.7%

I-HR, intermediate heterogeneity; HR, heteroresistance.

a *K. pneumoniae*: carbapenem-sensitive *K. pneumoniae*.

Interestingly, the ERV HR-positive rate, as determined by the PAP method, was significantly lower than those reported in other studies (Zhang et al., 2018; Zheng et al., 2018). Consequently, an alternative HR testing method was adopted. Prior research has highlighted the capability of the modified *E*-test method to detect bacterial HR (El-Halfawy and Valvano, 2015). Thus, 93 strains of bacteria were selected for modified E-tests, with the outcomes summarized in Table 3. The current study revealed that 18.3% of the strains were heteroresistant, with the proportions of CREC, eco-ESBL, MRSA, CRAB, and CRKP HR strains being 21.1, 15.0, 11.5, 30.0, and 28.6%, respectively. Nonetheless, HR-positive strains confirmed via the modified E-test method were not consistently replicated in the PAP test, indicating a degree of false positivity inherent to the modified *E*-test method.

**Table 3:** Detection of the heteroresistant phenotype by *E*-test.

Bacteria	Strains	Colonies in the inhibition zone	Rate of colonies in the inhibition zone (%)
CREC	19	4	21.1%
eco-ESBL	20	3	15.0%
MRSA	26	3	11.5%
CRAB	10	3	30.0%
CRKP	14	4	28.6%
*E. cloacae*	4	0	0.0%
Total	93	17	18.3%

### The ERV HR-CRKP can survive under ERV pressure

**Figure 2:**
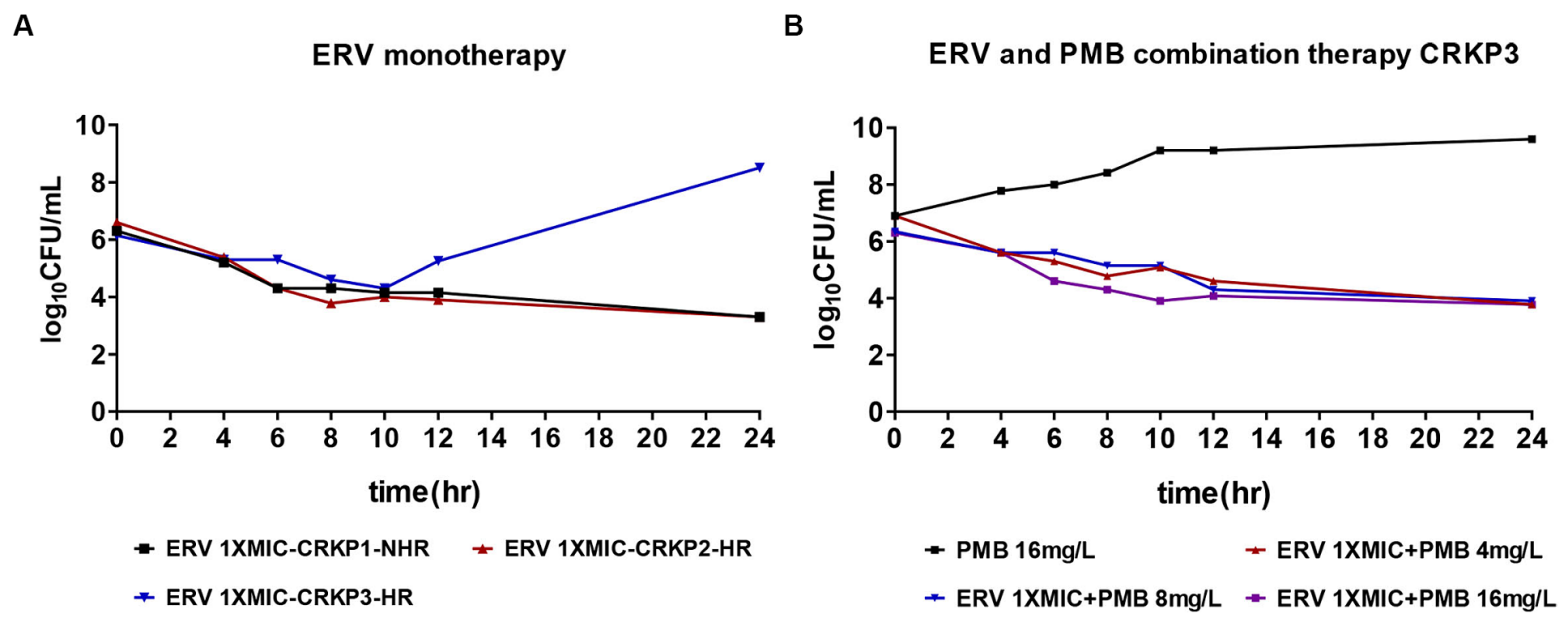
Time-killing assay for ERV and PMB monotherapy versus combination therapy against CR isolates. **(A)** ERV (1x MIC) as monotherapy. **(B)** ERV (1x MIC) in combination with varying concentrations of PMB (4, 8, and 16 mg/L). HR, heteroresistance; NHR, non-heteroresistant.

In order to determine the bactericidal effect of ERV against CRKP, experiments were conducted using an ERV concentration at 1x MIC. The results demonstrated that, in contrast to the non-heteroresistant (NHR) strains, one heteroresistant strain (CRKP3) exhibited an increase in total CFU after 10 h of ERV exposure (Figure 2A), signaling that heteroresistant strains could contribute to a diminished bactericidal effect of ERV *in vitro*. Combination therapy is frequently administered to circumvent issues related to antibiotic therapeutic failure in the clinical setting. Given the pivotal role of polymyxins in the therapeutic landscape for CRKP, the efficacy of ERV in conjunction with PMB against resistant CRKP3 subpopulations was investigated. Notably, compared to treatment with 16 mg/L PMB alone, dual treatment with ERV and PMB effectively inhibited the total CFU and simultaneously reduced the required PMB concentration (Figure 2B). Additional experiments with CRKP3 were conducted to elucidate the mechanisms behind the reduced bactericidal effect of ERV.

### ERV HR is unstable and transient

**Figure 3:**
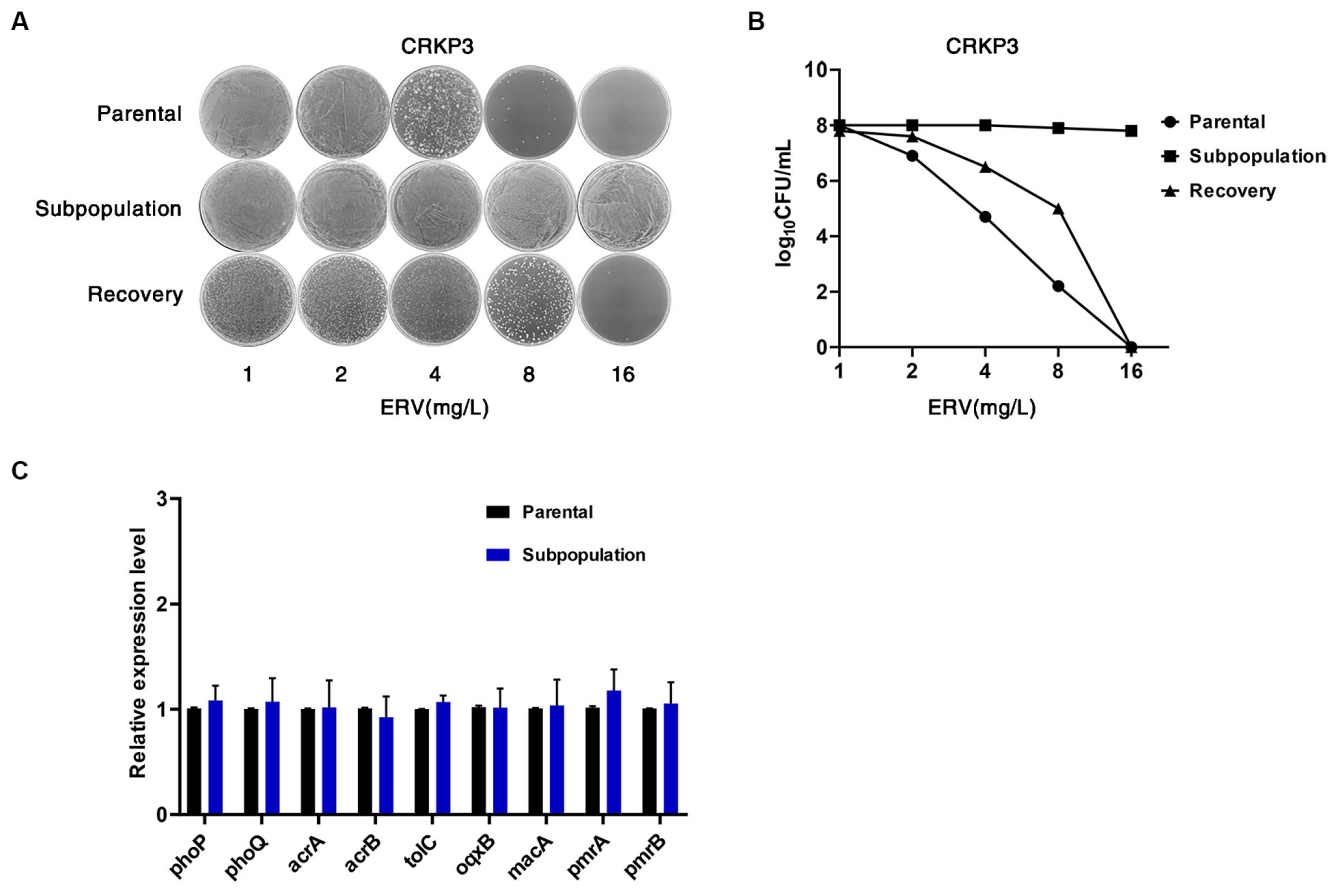
ERV HR is unstable and transient. **(A,B)** Resistance stability of CRKP3 parental bacteria, subpopulation, and recovery bacteria using the PAP method. **(C)** Expression analysis results for efflux components OqxB, MacA, AcrAB-TolC, and two-component systems PmrAB and PhoPQ system in ERV-heteroresistant strains (CRKP3) and the resistant subpopulation. Expression levels were determined by qRT-PCR and normalized against the *rpsL* housekeeping gene using the 2^−ΔΔCt^ CT method. Experiments were conducted in triplicate. A statistically significant difference is denoted by * (*p* < 0.05).

In order to explore the stable inheritance of the CRKP3 HR phenotype, antibiotic resistance stability experiments were performed. CRKP3 subpopulations were isolated on PAP plates at a 0.5x MIC concentration. Recovery bacteria derived from CRKP3 subpopulations were cultured for an additional 10 passages without selection pressure. PAP analysis revealed the absence of HR in both CRKP3 subpopulations and recovery bacteria, highlighting the transient and unstable nature of HR. In addition, the drug resistance level of CRKP3 subpopulations was significantly higher. Although the drug resistance level of the recovery bacteria was lower than that of the subpopulations, it was still higher than that of the parental strain, indicating that exposure to antibiotic stress may lead to the development of drug-resistant strains (Figures 3A,B).

Previous research has linked HR to the expression of bacterial efflux pump genes and two-component system genes (Zheng et al., 2018; Ma et al., 2019). In the current study, real-time PCR was performed to analyze the expression levels of the efflux components OqxB, MacA, AcrAB-TolC, and the two-component systems PmrAB and PhoPQ in the CRKP3 parental strains and subpopulations. Remarkably, compared with the parental strains, there were no significant differences (*p* > 0.05) in the expression of these efflux pump genes and the two-component systems in the resistant subpopulations (Figure 3C).

### Unstable resistance of subpopulations linked to fitness cost and unstable gene mutation

**Figure 4:**
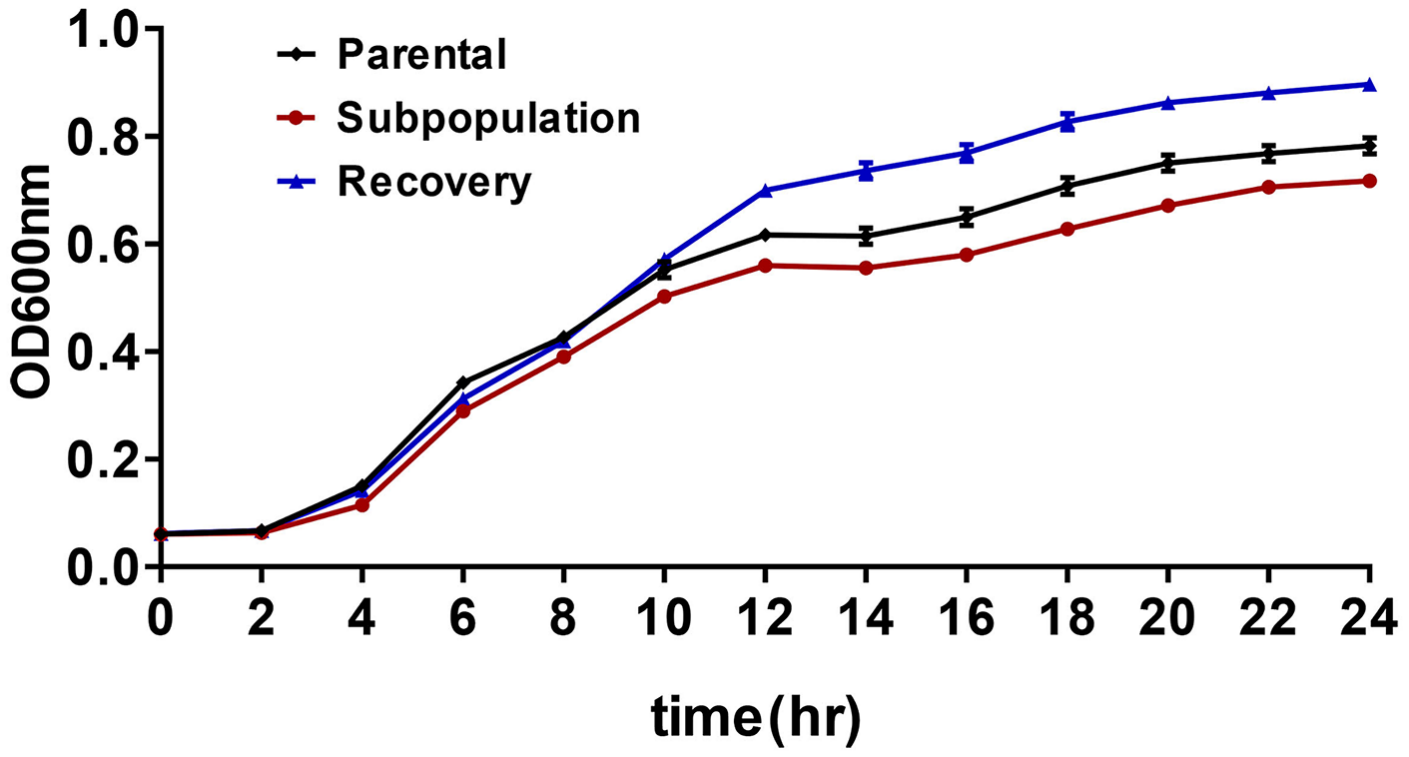
Fitness cost assessments of ERV-heteroresistant isolates (CRKP3). Growth kinetics of CRKP3 parental bacteria, subpopulations, and recovery bacteria were determined in the absence of selective pressure.

The fitness cost associated with CRKP3 was evaluated by charting the growth trajectories of the parental strain, subpopulations, and recovery bacteria. The growth rate of the CRKP3 subpopulations was lower than that of the parental strain and the recovery bacteria, implying a fitness cost associated with drug resistance acquisition in the CRKP3 subpopulations (Figure 4).

WGS analysis of the CRKP3 parental strain, its subpopulation, and the recovery strain was conducted to identify the mechanisms responsible for the unstable HR phenotype. Different nucleotide and resultant amino acid alterations are summarized in Supplementary Table S2. Interestingly, the subpopulation exhibited a mutation in the *vgrG* gene (Pro 788 Leu), not present in the parental or recovery strains. The role of *vgrG* in antibiotic resistance has been previously explored (Wang et al., 2018). Although mutations were observed in other genes, their contribution to ERV HR remains to be clarified.

## Discussion

ERV is anticipated to be a promising treatment option for CRKP infections. To the best of our knowledge, this is the first report on ERV HR in clinically common pathogens, particularly those resistant to carbapenems. As previously documented, a potential obstacle in the clinical application of ERV is HR (Zheng et al., 2018). Since its discovery in 1970, HR has been detected across a broad spectrum of antibiotics, encompassing beta-lactams, aminoglycosides, quinolones, tetracyclines, and colistin (Moon et al., 2022; Roch et al., 2023). For instance, a study examining colistin HR in highly resistant CRE in the United States from 2012 to 2015 evinced that colistin HR was present in 10.1% (41/408) of the isolates, surpassing conventional resistance, which was at 7.1% (29/408) (Band et al., 2021). In the current research, the PAP method was employed to investigate the prevalence of ERV HR in clinical isolates from two centers. The results showed that the ERV HR rate was not high: 2 strains were heteroresistant (0.7%, 2/280), while 68 strains exhibited intermediate heterogeneity (24.3%, 68/280). Furthermore, a modified E-test was used as an alternative screening method for HR. Testing 93 strains with this method identified an HR strain proportion of 18.3%. Hence, results from the modified *E*-tests could not be replicated by PAP, aligning with previous reports (Nicoloff et al., 2019). The discrepancies between the two screening techniques could arise from methodological differences, variations in bacterial density, and differences in ERV concentrations, contributing to the inconsistent detection of bacterial HR subpopulations. With some false-positive rates observed in modified *E*-tests, PAP results were considered more reliable than those from modified *E*-tests. Based on the findings presented herein and those of previous research (Nicoloff et al., 2019), the use of the PAP method for HR detection is recommended.

The present study demonstrated that HR could lead to a diminished bactericidal effect of ERV *in vitro*. Treating CRKP with 1x MIC of ERV *in vitro* led to a marked increase in the total CFU after 10 h, followed by a resurgence of the original bacterial population in subsequent hours. Prior research has highlighted instances where HR led to antibiotic treatment failure (Band et al., 2016, 2018). For example, despite clinical CRKP strain isolates being labeled as colistin-susceptible, colistin treatment failed to rescue mice infected by HR strains (Band et al., 2018). Similarly, *E. cloacae* isolates have been linked to colistin treatment failure (Band et al., 2016). These examples suggest that the challenge of clinically detecting a few resistant subpopulations might be the cause of HR-related treatment failure. These resistant subpopulations often proliferate rapidly to become the dominant population in the presence of antibiotics, leading to antibiotic treatment failure. Consequently, there is an urgent need for novel therapeutic strategies capable of inhibiting the growth of ERV-resistant subpopulations. Previous literature has underscored the potential advantages of combination therapies in the management of CR strains (Peck et al., 2012; Tascini et al., 2013). The present study confirmed that a low-dose antibiotic combination (1x MIC ERV with 4 mg/L PMB) significantly reduced bacterial CFU, suggesting that combination therapy may inhibit the emergence of resistant subpopulations and reduce the necessary dose of antibiotics.

The HR phenotype is generally unstable, and when detected in clinical isolates, it rapidly reverts to susceptibility in the absence of antibiotic pressure. In this study, the HR phenotype was identified in the CRKP3 parental strain but disappeared after the strain underwent 10 passages in the absence of ERV. Such transient characteristics pose challenges and elevate the costs associated with clinical detection. To probe the mechanisms underlying the instability of the HR phenotype, real-time PCR analyzed the expression levels of efflux components OqxB, MacA, and AcrAB-TolC, and the two-component systems PmrAB and PhoPQ in the CRKP3 parental strains and subpopulations. Prior studies have linked these genes to ERV HR or antibiotic HR in *K. pneumoniae* (Zheng et al., 2018; Ma et al., 2019). The current study found comparable expression levels of these genes between the CRKP3 parental strains and subpopulations, indicating the existence of other mechanisms for ERV HR. WGS analysis of the CRKP3 parental strain, resistant subpopulation, and recovery bacteria revealed several non-synonymous mutations in the genes encoding *vgrG*, hypothetical protein, *LysB* family phage lysis regulatory protein, lysozyme, DNA-binding ATP-dependent protease La, baseplate assembly protein, and phage-associated proteins. These mutations might be associated with ERV HR, although further investigation is needed to confirm the mechanisms of ERV HR and the association of mutations, particularly in the *vgrG* gene, with ERV HR. Point mutations, IS, or small deletions in the *ubiJ* and *cydA* genes have been reported to induce small colony variant phenotypes and aminoglycoside resistance and are also associated with aminoglycoside HR in *E. coli*, *K. pneumoniae*, and *Salmonella typhimurium* (Nicoloff et al., 2019). Fitness cost assessments showed that resistant subpopulations incurred a partial growth cost to acquire resistance. Interestingly, recovery bacteria grew faster than the parental strain, challenging the typical observation that fitness cost is observed as a reduced bacterial growth rate. Several *in vitro* studies (Björkman et al., 1998, 2000; Nagaev et al., 2001) have demonstrated that, in most cases, compensation can be detected when resistant bacteria are serially passaged, with the extent of recovery and the type and number of compensatory mutations depending on the specific bacterial and resistance mechanisms and the environmental conditions in which compensation occurs. Thus, under certain conditions, fitness costs are compensated by increased bacterial growth rates. These findings underscore that multigene variations and fitness costs are essential factors in the development of ERV HR in CRKP. The data presented in the study are deposited in the National Center for Biotechnology Information (NCBI), accession number PRJNA1086703.

In conclusion, the current study delineates the prevalence of ERV HR in common clinical pathogens. HR led to a reduced bactericidal effect in CRKP following ERV exposure. Further analysis revealed the unstable and transient nature of ERV HR in CRKP, likely attributable to fitness costs and gene mutations. The present study had several limitations, including the limited sample size of HR screening, which yielded a small number of strains confirmed as HR. Additionally, as this study focused on the bactericidal effect of ERV *in vitro*, there is a pressing need for *in vivo* investigations. Further investigations are warranted to explore the mechanisms underlying HR in pathogenic bacteria to effectively counter antibiotic resistance.

## Data availability statement

The data presented in the study are deposited in the National Center for Biotechnology Information (NCBI), accession number PRJNA1086703.

## Author contributions

YZ: Visualization, Validation, Methodology, Investigation, Formal analysis, Data curation, Conceptualization, Writing – original draft, Writing – review & editing. DL: Project administration, Validation, Supervision, Resources, Investigation, Funding acquisition, Writing – review & editing. YonL: Supervision, Resources, Project administration, Methodology, Writing – review & editing. QL: Resources, Project administration, Visualization, Methodology, Formal analysis, Writing – review & editing. HL: Methodology, Writing – review & editing, Software, Formal analysis, Data curation, Supervision. PZ: Validation, Software, Data curation, Writing – review & editing, Visualization, Methodology, Formal analysis. YaqL: Writing – review & editing, Project administration, Methodology, Formal analysis, Conceptualization, Supervision. LC: Data curation, Supervision, Resources, Project administration, Funding acquisition, Writing – review & editing. WY: Methodology, Supervision, Resources, Project administration, Data curation, Writing – review & editing. YanL: Project administration, Visualization, Supervision, Resources, Methodology, Funding acquisition, Formal analysis, Data curation, Conceptualization, Writing – review & editing.
