# Supplementary material for: Detection and characterization of eravacycline heteroresistance in clinical bacterial isolates
Source: Front Microbiol. 2024 Mar 27;15:1332458. doi: 10.3389/fmicb.2024.1332458 (PMC11004243; doi:10.3389/fmicb.2024.1332458)
Supplement: Supplementary file 1 [file Table_1.docx]

**Table S1 Sequences of qPCR Primers.**

| Name | Sense primer (5'-3') | Antisense primer (5'-3') | Source |
| --- | --- | --- | --- |
| *phoP* | GCGTCACCACCTCAAAGTTC | GGCGATATCCGGGAGATGTT | This work |
| *phoQ* | CTCAAGCGCAGCTATATGGT | TCTTTGGCCAGCGACTCAAT | This work |
| *acrA* | ATGTGACGATAAACCGGCTC | CTGGCAGTTCGGTGGTTATT | This work |
| *acrB* | CGATAACCTGATGTACATGTCC | CCGACAACCATCAGGAAGCT | This work |
| *tolC* | CTACAAACAGGCGGTGGTCT | TGTTCAGCTCGTTGATCAGG | This work |
| *oqxB* | ATCAGGCGCAGGTTCAGGT | CGCCAGCTCATCCTTCACTT | This work |
| *macA* | TATGAAGGTAAACTGAAAGACA | GAAGCGGGCATAATAGAA | This work |
| *pmrA* | GATGAAGACGGGCTGCATTT | ACCGCTAATGCGATCCTCAA | This work |
| *pmrB* | TGCCAGCTGATAAGCGTCTT | TTCTGGTTGTTGTGCCCTTC | This work |
| *rpsL* | CCGTGGCGGTCGTGTTAAAGA | GCCGTACTTGGAGCGAGCCTG | This work |
